# The role of radiotherapy in pelvic nodal recurrence following definitive treatment for prostate cancer

**DOI:** 10.1097/MOU.0000000000001322

**Published:** 2025-07-17

**Authors:** Alessandro Dematteis, Marcin Miszczyk, Angelo Cormio, Akihiro Matsukawa, Paolo Gontero, Shahrokh F. Shariat

**Affiliations:** a Department of Urology, Comprehensive Cancer Center, Medical University of Vienna, Vienna, Austria; b Division of Urology, Department of Surgical Sciences, AOU Città della Salute e della Scienza at Molinette Hospital and University of Turin, Turin, Italy; c Division of Anatomy, Medical University of Vienna, Vienna, Austria; d Collegium Medicum, Faculty of Medicine, WSB University, Dąbrowa Górnicza, Poland; e Department of Urology, Azienda Ospedaliero-Universitaria Ospedali Riuniti Di Ancona, Università Politecnica Delle Marche, Italy; f Department of Urology, The Jikei University School of Medicine, Tokyo, Japan; g Department of Urology, Semmelweis University; h Centre for Translational Medicine, Semmelweis University, Budapest, Hungary; i Department of Urology, University of Texas Southwestern, Dallas, Texas, USA; j Department of Urology, Second Faculty of Medicine, Charles University, Prague, Czech Republic; k Hourani Center for Applied Scientific Research, Al-Ahliyya Amman University, Amman, Jordan; l Karl Landsteiner Institute of Urology and Andrology, Vienna, Austria; m Research Center for Evidence Medicine, Urology Department, Tabriz University of Medical Sciences, Tabriz, Iran

**Keywords:** local, neoplasm recurrence, pelvic radiotherapy, PET, prostatic neoplasm, salvage therapy

## Abstract

**Purpose of review:**

To summarize recent evidence on the role of radiotherapy in managing pelvic lymph node (PLN) recurrence following curative-intent primary therapy for prostate cancer (PCa), focusing on radiotherapy strategies, novel medical imaging, and oncological outcomes.

**Recent findings:**

Prostate-specific membrane antigen PET (PSMA-PET) has improved accuracy of staging in patients with PCa; however, more often than not, it fails to correctly identify PLN metastases, and the impact on clinical outcomes of the patients is uncertain. Metastasis-directed therapies (MDT) combined with short-term androgen-deprivation therapy (ADT) in patients with PLN recurrence are associated with a significantly higher risk of recurrence compared to more comprehensive approaches. Emerging data support the role of elective nodal radiotherapy (ENRT) combined with short-term androgen deprivation therapy (ADT) and radiotherapy boost to the PLN metastases to enhance disease control. Notably, despite treating a more extensive pelvic region than MDT, ENRT does not appear to significantly increase acute toxicity or negatively impact quality of life (QoL). Recent evidence suggests a role for androgen receptor pathway inhibitors (ARPI), such as enzalutamide, in patients with high-risk biochemical recurrence, introducing a new treatment paradigm for patients ineligible for salvage radiotherapy. Ongoing prospective studies are refining the role of radiotherapy in combination with systemic treatments.

**Summary:**

Despite PSMA-PET allowing for improved staging and better patient-tailored decisions, patients with PLN recurrence continue to benefit from comprehensive multimodal treatment approach. Elective PLN irradiation combined with radiotherapy boost and ADT lead to improved disease control, without compromising safety and toxicity. ARPI+ADT and ARPI-monotherapy emerge as alternatives for select patients.

## INTRODUCTION

A significant number of patients experience biochemical recurrence (BCR) within 5–10 years of definitive treatment for prostate cancer (PCa), with rates reaching up to 50% in patients with very high-risk disease [1–4]. While BCR is associated with an increased risk of distant metastasis and PCa-specific mortality [5], excessive treatment can lead to unnecessary toxicity [6,7]. The advent of novel molecular imaging techniques, in particular PSMA-PET, has transformed disease detection, allowing earlier identification of regional and distant recurrences beyond the limits of conventional imaging [8–10]. However, whether this improves patient outcomes in the setting of node-positive disease remains uncertain. 

**Box 1:**
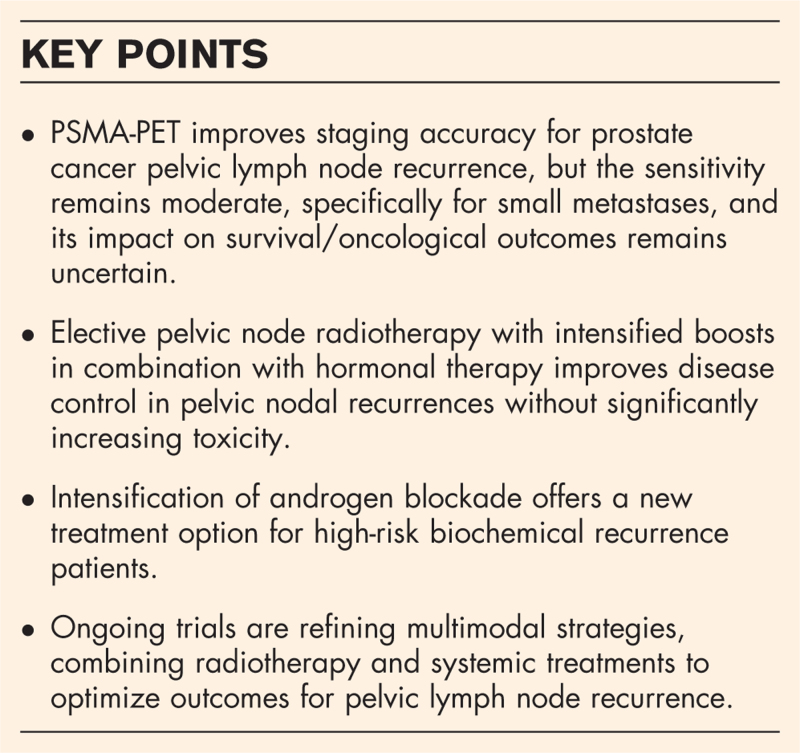
no caption available

Isolated pelvic nodal recurrence, observed in approximately 30% of patients with BCR postradical prostatectomy, has become a key therapeutic focus due to its frequency and clinical implications [9,11]. Current guidelines recommend salvage radiotherapy (sRT) together with androgen deprivation therapy (ADT) for nodal recurrence [2,12,13]; yet the role of locoregional salvage treatments remains debated, pending stronger prospective evidence [14]. Recent studies have evaluated salvage lymph node dissection (LND) and radiotherapy in selected oligorecurrent cases, challenging traditional approaches and seeking to defer systemic therapy with its associated decrement in quality of life (QoL) [15,16]. Metastasis-directed therapy (MDT), including stereotactic body radiotherapy (SBRT), alone or in addition to elective nodal radiotherapy (ENRT), offers a potential targeted approach to enhance local control, delay disease progression and subsequent toxicity of salvage systemic treatments [17]. While MDT shows promise, its sole use remains limited to carefully selected patients within clinical studies. This review examines recent advancements in radiotherapy for pelvic nodal recurrence of PCa, aiming to provide specialists with evidence-based, personalized strategies while addressing uncertainties in patient selection and decision-making. Table 1 provides a summary of the prospective studies included.

**Table 1:** Prospective studies included

Study, author	Type of study, accrual period	Population	Imaging technique for MTS detection	Intervention arms, number of patients	Primary, secondary endpoints
PLATIN-4 Fink *et al.*, 2023	Single-arm, bicentric, phase 2 trial (2009–2018)	PCa recurrence after RP, and clinical suspicion for pelvic nodal recurrence	Conventional PSMA PET/CT (31%)	PBRT+ENRT+SIB+ADTTotal: 39	RT safety and feasibility PFS and OS
PLATIN-5 Fink *et al.*, 2023	Single-arm, bicentric, phase 2 trial (2009–2018)	PCa recurrence after RP and PBRT, and clinical suspicion for pelvic nodal recurrence	Conventional PSMA PET/CT (59%)	ENRT+SIB+ADT Total 39	RT safety and feasibility PFS and OS
NRG Oncology/RTOG 0534 SPPORT Pollack *et al.*, 2022	Three-arm, multicenter, phase 3 RCT (2008–2015)	PSA persistency of recurrence after RP. No clinical/pathological nodal MTS	Conventional	PBRT: 564 PBRT+STADT: 578 PBRT+ENRT+STADT: 574 Total: 1716	FFP at 5 years (freedom from progression) OS and toxicity
PEACE V-Storm Ost *et al.*, 2025	Two-arm, multicenter, phase 2 RCT (2018–2021)	Up to five pelvic nodal PCa recurrences after radical local treatment	Choline or PSMA PET/CT-MRI	MDT+STADT: 97 ENRT+MDT+STADT: 93 Total: 190	MFS BRFS, toxicity and QoL
EMBARK Freedland *et al.*, 2023	Three-arm, multicenter, phase 3 RCT (2015–2018)	High risk BCR after local treatment for PCa	Conventional imaging	ENZ+ADT: 355 ENZ: 355 ADT: 358 Total: 1068	MFS BRFS, OS, time to new antineoplastic therapy
OLIGOPELVIS (GETUG-P07) Vaugier *et al.*, 2024	Single-arm, multicenter, phase 2 trial (2014–2016)	Up to 5 pelvic nodal PCa recurrences after local treatment	Choline PET/CT	ENRT+SIB+STADT Total: 67	PFS BRFS, OS, TFS, ADT-FS, toxicity, QoL
Prospective trial Fodor *et al*., 2023	Single-arm, single-center, phase 2 trial 10-year follow-up (2009–2015)	PCa BCR after previous treatments (RP, RT, systemic, and combined) and nodal relapse only.	Choline PET/CT	ENRT+SIB ± ADT Total: 60	BRFS, MFS, CRFS, OS, toxicity
NCT03368547- NCT02611882-NCT02919111 Hope *et al.*, 2021	Single-arm, bicentric, phase 3 imaging trial (2018–2021)	Intermediate- to high-risk PCa patients eligible to undergo RP	PSMA PET/CT	PSMA PET/CT Total: 764	Diagnostic accuracy for PCa pelvic nodal MTS.
PSMA-SRT NCT03582774 Armstrong *et al.*, 2024	Two-arm, bicentric, phase 3 RCT (2018–2020)	PCa BCR after RP	PSMA PET/CT	sRT without PET: 90 PET prior to sRT planning: 103 Total: 193	BRFS (not yet available) Impact of PET on sRT planning
GETUG-AFU 16 Carrie *et al.*, 2019	Two-arm, multicenter, phase 3 RCT (2006–2010)	PCa BCR after RP, without clinical disease.	Conventional imaging	RT+ADT: 369 RT: 374 Total: 743	PFS MFS, OS, toxicity, QoL
RADICALS-HD Parker *et al.*, 2024	Two-arm, multicenter, phase 3 RCT (2008–2015)	Patients due for RT at any time after RP for PCa	Conventional imaging	STADT+RT: 761 LTADT+RT: 762 Total: 1523	MFS OS, ADT-FS, CPFS, toxicity, QoL
SALV-ENZA Tran *et al.*, 2023	Two-arm, multicenter, phase 2 RCT (2015–2020)	PCa BCR after RP, without MTS	Conventional imaging	ENZ+PBRT: 43 RT: 43 Total: 86	FFPP Local recurrences, MFS, safety, and feasibility.
FORMULA 509 Nguyen *et al.*, 2023	Two-arm, multicenter, phase 2 RCT (2017–2020)	PCa high-risk BCR after RP	Conventional imaging	ABI+APA+STADT+sRT (±ENRT): 173 BIC+STADT+RT(±ENRT): 172 Total: 345	PPFS MFS

ABI, abiraterone; ADT, androgen deprivation therapy; APA, apalutamide; BIC, bicalutamide; BRFS, biochemical recurrence-free survival; BT, brachytherapy; CRFS, clinical relapse-free survival; ENRT, elective nodal radiotherapy; ENZ, enzalutamide; FFPP, freedom from prostate-specific antigen progression; LTADT, long-term androgen deprivation therapy (24 months); MFS, metastasis-free survival; MTS, metastases; OS, overall survival; PBRT, prostate bed radiotherapy; PFS, progression-free survival; PPFS, PSA progression-free survival; PSA, prostate-specific antigen; PSMA-PET/CT, prostate-specific membrane antigen PET/computed tomography; RCT, randomized controlled trial; RP, radical prostatectomy; RT, radiation therapy; SIB, simultaneous integrated boost; sRT, salvage radiotherapy; STADT, short-term androgen deprivation therapy (6 months).

## DIFFERENT RADIOTHERAPY APPROACHES TO PELVIC RECURRENCES AND ASSOCIATED TOXICITY

There is no universally accepted radiation strategy for isolated pelvic lymph node (PLN) recurrences in PCa. Several locoregional treatment options have been introduced in clinical practice, differing in the extent of treated fields, delivery techniques, and fractionation schedule. The planning of radiotherapy depends on the perceived balance between the risk of distant progression and the potential toxicity of local therapy [18]. Recent studies have investigated a spectrum of radiotherapy techniques, ranging from the focal SBRT to the broader whole pelvis radiotherapy (WPRT), to evaluate their feasibility and clinical outcomes.

### Elective nodal radiotherapy and prostate bed radiotherapy

The PLATIN-4 and PLATIN-5 trials [19] examined patients with PLN recurrence after radical prostatectomy alone (*n* = 39) or radical prostatectomy followed by prostate bed radiotherapy (PBRT; *n* = 39). Patients received ENRT with a boost to involved nodes (PLATIN-5) and the prostate bed (PLATIN-4), alongside long-term ADT. The treatment was safe and feasible, and late grade 3 or higher genitourinary and gastrointestinal (GI) toxicities occurred only in 4% of patients. Median progression-free survival (PFS) was 66 months in PLATIN-4 and 39 months in PLATIN-5, suggesting that this approach can lead to durable cure.

The SPPORT trial [20], was the first randomized controlled trial (RCT) to explore the benefits of ENRT in patients with detectable PSA levels after radical prostatectomy. Eligible patients (*n* = 1716) were randomized to: PBRT alone (arm 1; *n* = 564), PBRT along with short-term ADT (4–6 months) (arm 2; *n* = 578), or both ENRT and PBRT with short-term ADT (arm 3; *n* = 574). Whil‘e this trial was not performed particularly in the N+ setting, the results demonstrate that extending radiotherapy to pelvic nodes in combination with short-term ADT significantly reduces disease progression compared to PBRT alone [hazard ratio: 0.50, 97.5% confidence interval (CI) 0.39–0.64], and could reduce the morbidity associated with subsequent salvage ADT, which is often a life-long management approach. Interestingly, the only significant increase in toxicity regarded late grade 2 or higher blood/bone marrow events, but the pattern was not consistent; the odds were significantly increased comparing patients treated with PBRT+ADT+/−ENRT [odds ratio (OR) 2.6, 95% CI 1.23–5.47, *P* = 0.006), but not PBRT+/−ENRT (OR 1.22, *P* = 0.26). These findings suggest that ENRT, even when administered after prior PBRT, is a viable and potentially curative option for nodal recurrence post-radical prostatectomy, offering acceptable rates of high-grade toxicity when paired with ADT.

### Hemi-pelvis versus whole pelvis radiotherapy

Trapp *et al.* [21] evaluated Hemi-pelvis radiotherapy (HPRT) in patients with PSMA-positive nodal recurrences post-radical prostatectomy in a retrospective multicenter study employing propensity score matching, finding that HPRT is associated with similar oncological outcome comparable to whole pelvis radiotherapy (WPRT) for nodal recurrence following radical prostatectomy. Key predictors of BRFS included concomitant ADT, radiation dose, and the use of an radiotherapy boost to PSMA-positive PLN. However, while this study generates an interesting hypothesis, it has significant limitations: authors acknowledge that not all known clinical variables were balanced between groups, and there are potentially important characteristics that have not been accounted for, such as the number and location of suspected PLN and presence of high-risk pathologic/biologic factors such as lymphovascular invasion [4,22,23].

### Stereotactic body radiotherapy to the lymph node metastases

Recent findings from the PEACE V-STORM trial [24^▪▪^] addressed oligorecurrent nodal PCa. In this phase 2 trial patients with PLN oligorecurrence (≤5 nodes) identified on PET/CT (83% PSMA, 17% choline) after radical therapy for PCa were randomized to either MDT (*n* = 97), or ENRT+MDT (*n* = 93), both arms receiving 6 months of ADT. Surgery was permitted as a form of MDT to the node in place of SBRT, though it was seldom utilized. At a median follow-up of 50 months, ENRT significantly improved 4-year metastasis-free survival (MFS) (76 versus 63%; hazard ratio 0.62, 80% CI 0.44–0.86; *P* = 0.063) compared to MDT alone. Grade at least 2 gastrointestinal toxicity was low in both arms: 5.3% of patients after SBRT and 6.6% after ENRT over 2 years. Grade at least 2 genitourinary toxicity occurred in 22.2% (SBRT) and 26.5% (ENRT), with more persistent symptoms in the ENRT group. Notably, PBRT was more frequent in the ENRT arm (41 versus 25%) and independently increased the risk of genitourinary toxicity (hazard ratio 0.55). Despite the broader ENRT field, toxicity profiles and QoL remained comparable between the two arms.

The OLIGOPELVIS (GETUG-P07) phase 2 single-arm trial [25^▪▪^] enrolled 67 men with five or less nodal recurrences identified on [18F]-fluorocholine PET/CT. This study assessed the efficacy of ENRT with a moderately hypofractionated simultaneous integrated boost (SIB) and 6 months of ADT. After 5 years of follow-up, pelvic sRT was well tolerated, with grade 2 or higher genitourinary toxicity reported in 13% of patients, comparable to rates in similar studies, but higher than those observed with SBRT alone [26]. Approximately 30% of patients remained disease-free, and 64% were free from ADT. These long-term results suggest that ENRT combined with short-term ADT offers sustained tumor control with limited toxicity in patients with oligorecurrent PLN detected via molecular imaging.

In summary, a range of locoregional well tolerated radiotherapy strategies, including WPRT, HPRT, ENRT, SBRT, and PBRT, often combined with ADT, offers diverse options for managing limited pelvic nodal recurrences. Nevertheless, definitive radiotherapy treatment recommendations remain uncertain due to the heterogeneity of approaches and the need for further high-quality evidence.

## IMAGING APPROACHES BEFORE PROSTATE-SPECIFIC MEMBRANE ANTIGEN-PET INTRODUCTION

Before the integration of PSMA-PET into clinical practice for PCa recurrences, conventional imaging or alternative molecular scan modalities were employed to detect regional PCa foci. A 2015 systematic review by Ploussard *et al.* [27] highlighted choline-PET as the reference imaging technique for detecting nodal relapses across all included studies. In this section of the manuscript, we review recent literature on imaging approaches distinct from PSMA-PET, including conventional techniques (CT, MRI, bone scan), and choline-PET.

### Conventional imaging

In the previously discussed SPPORT trial [20], CT, MRI, and bone scan were utilized to rule out distant metastases in patients with PSA detectable following radical prostatectomy. The trial suggested that extending PBRT to include pelvic nodes, combined with ADT, improves oncological outcomes.

Similarly in the EMBARK trial [28^▪▪^] a phase 3 study involving patients with high-risk biochemical recurrence (BCR) after definitive treatment, patients were excluded if conventional imaging (CT, MRI, and bone scan) revealed distant metastases or if they were considered eligible for sRT. Participants were randomized in a 1 : 1:1 ratio to receive enzalutamide+ADT, placebo+ADT, or enzalutamide monotherapy. The results indicated that enzalutamide combined with ADT or as monotherapy significantly prolonged MFS compared to ADT alone. Notably, EMBARK relied solely on conventional imaging, with no mention of PET tracers.

As with the SPPORT trial, the absence of nuclear imaging could influence the applicability of its findings to contemporary salvage therapy recommendations.

### Choline-PET

The already mentioned OLIGOPELVIS (GETUG-P07) trial identified nodal recurrences through [18F]-fluorocholine-PET.

In a prospective single-arm study [29^▪^], [11C]-choline-PET was used to select 60 men with BCR and positive lymph nodes only. Participants received ENRT with SIB targeting the positive nodes; 57% had PLN involvement alone, 43% had para-aortic positive nodes, and 48% received ADT. To date, this is the study with the longest follow-up (10 years) to evaluate ENRT in combination with PET-guided SIB for PCa nodal recurrence. This approach demonstrated good outcomes, with a median castration resistance-free survival (CRFS) of 67 months and a median overall survival (OS) of 110 months, underscoring the efficacy of choline-guided ENRT with SIB in this setting.

In the above presented PEACE V-STORM trial, 17% of participants underwent choline-PET, while the remaining 83% were assessed with PSMA-PET for PLN recurrence.

Both choline and fluciclovine imaging tracers showed promising results in patients with BCR, earning recommendations in international guidelines [2]. However, their diagnostic accuracy is lower when compared to PSMA-PET, particularly at low PSA levels [30–32].

## PROSTATE-SPECIFIC MEMBRANE ANTIGEN-PET

In the context of BCR following definitive treatment, PSMA-PET stands out as the imaging modality with the highest accuracy, particularly at low PSA levels. Its ability to localize recurrences plays a role in planning salvage treatments. International guidelines recommend PSMA-PET for relapse after radical prostatectomy or definitive radiotherapy when the results are likely to influence subsequent therapeutic decisions [2,12,33]. Notably, PSMA-PET demonstrates moderate accuracy in detecting nodal metastases in men with intermediate- to high-risk PCa prior to radical prostatectomy, as shown by Hope *et al.* [34] in a phase 3 diagnostic efficacy trial. The authors reported a sensitivity of 0.40 and a specificity of 0.95 for nodal detection. Although this sensitivity is lower than previously reported studies, the high specificity suggests that a positive PSMA PET strongly indicates nodal metastasis. Conversely, the negative-predictive value was 0.81, meaning that approximately 20% of intermediate-risk to high-risk patients with a negative scan may still harbor nodal disease. Therefore, PSMA-PET, while valuable, often fails to reliably identify nodal metastases.

The impact of PSMA-PET on clinical decision-making in PCa recurrences was evaluated in a prospective randomized phase 3 trial by Armstrong *et al.* [35^▪^]. In this study, 193 patients with BCR post-radical prostatectomy were assigned to either immediate sRT (control arm, *n* = 90) or PSMA-PET prior to sRT (investigational arm, *n* = 103). Over one-third of patients in the investigational arm experienced a management change based on imaging findings. For instance, PSMA-PET optimized patient selection for sRT, prompted adjustments to radiotherapy fields and doses, and influenced the use of systemic therapy. The primary endpoint, biochemical recurrence-free survival (BRFS) following sRT, is pending final analysis in 2025, leaving open question whether these management changes translate into improved oncological outcomes.

Recently, Holzgreve *et al.* [36] conducted a retrospective cross-sectional study of 182 patients from four prospective trials to assess the role of PSMA-PET in patients eligible for the EMBARK trial (high-risk BCR with negative conventional imaging). Their findings challenge the interpretation of prior studies, including EMBARK itself and SPPORT. PSMA-PET identified pelvic nodal recurrences in 29% of patients overall, with nodal metastases (N1 and/or M1a) detected in 44% post-radical prostatectomy, 15% postdefinitive radiotherapy, 44% post-sRT and radical prostatectomy, and 38% across all groups. Distant metastases were observed in 46% of patients, with polymetastatic disease (at least five lesions) in 24%. These results suggest that conventional imaging significantly understages disease, highlighting the diagnostic superiority of molecular imaging.

These findings raise questions about patient eligibility for MDT, either as an adjunct to or a precursor of systemic therapy. In a commentary, Eistein *et al.* [37] assessed the implications of the EMBARK trial for choosing between MDT, sRT, or enzalutamide in the setting of BCR, weighing MFS against treatment-free survival. In conclusion, prospective studies integrating PSMA-based risk stratification with patient outcomes are essential to guide future management.

With PSMA-PET isolated PLN recurrences post-radical prostatectomy are increasingly identified, prompting debate about the safety of omitting PBRT to reduce local toxicity. Challis *et al.* [38] conducted a prospective study of 46 patients with PSMA-PET only nodal relapse post-radical prostatectomy, divided into PB+ENRT (*n* = 22) and ENRT-only (*n* = 24) cohorts. All patients were recommended ADT, with PBRT avoidance based on PCa characteristics and determined by the radiation oncologist and a multidisciplinary team. The results confirmed reduced toxicity with PBRT omission, consistent with existing literature. Moreover, the 4-year BRFS of 64% in the ENRT group aligned with the 4-year BRFS of 57% from the above-mentioned PEACE V-STORM trial [24^▪▪^]. This suggests that for carefully selected patients with PSMA-detected only nodal relapse, ENRT alone may be a viable strategy. However, a limitation is the reliance on PSMA-PET sensitivity, which may miss recurrences below its detection threshold [34].

Alternative MDT strategies guided by PSMA-PET have also been assessed. A systematic review and pooled analysis [39] compared oncological outcomes of salvage LND versus sRT for PET-positive nodal relapses after radical prostatectomy, encompassing 19 studies (995 men in the LND group, 1481 in the sRT group). Despite significant heterogeneity, pooled rates of systemic, imaging, and PSA progression were higher with LND than sRT. These findings underscore the importance of considering treatment failure risks when planning focal salvage treatment, to ensure more appropriate decision-making and patient counseling.

For patients with BCR following radical prostatectomy and a negative PSMA-PET, optimal management remains under debate. The EAU guidelines recommend early sRT, despite negative findings at molecular imaging [2]. Adebahr *et al.* [40] conducted a multicenter retrospective study involving 300 patients with BCR and negative PSMA-PET. Among them, 253 received PBRT, 46 additional ENRT, and only 41 concomitant ADT. The 3-year BRFS was 77.5 and 48.3% for patients with PSA levels before PSMA-PET 0.5 ng/ml or less and greater than 0.5 ng/ml, respectively; findings that align with prior studies in PSMA-PET positive cohorts [9,41]. ENRT did not significantly improve BRFS, possibly due to the limited number of patients receiving nodal boosts. After sRT, isolated pelvic nodal recurrence was the predominant relapse pattern. These results support the use of early sRT in case of BCR with negative molecular imaging, while emphasizing the need for randomized controlled trials before omitting sRT in these patients, and highlight the possibility that PSMA-PET may have missed nodal metastases, as suggested by the relapse patterns observed after salvage radiotherapy.

In the era of PSMA-PET, its superior diagnostic accuracy has revolutionized the detection and management of BCR, guiding tailored salvage strategies, though oncological benefits of these refined approaches still need to be confirmed.

## SYSTEMIC TREATMENT CONSIDERATIONS

Combining ADT with sRT in the setting of PCa recurrence following definitive treatment and its impact on oncological outcomes remains a subject of active investigations. Recent prospective studies in BCR setting have provided compelling evidence supporting the efficacy of this combined approach. The previously discussed SPPORT trial [20], demonstrated the benefit of adding ADT to sRT, resulting in improved progression-free survival (ENRT+PBRT+ADT versus PBRT alone: hazard ratio 0.50, 97.5% CI 0.39–0.64; PBRT+ADT versus PBRT alone: hazard ratio 0.6, 97% CI 0.47–0.77). In the SPPORT trial, participants were eligible if there was no pathologic or radiographic (conventional imaging) evidence of lymph node involvement. Similarly, the GETUG-AFU 16 trial [42] in which men with BCR after radical prostatectomy were randomized to either sRT alone or sRT with short-term LHRH agonist showed that 6 months ADT significantly reduced the risk of disease progression (120-months PFS: hazard ratio 0.54, 95% CI 0.43–0.68) and mortality (OS: hazard ratio 0.77, 95% CI 0.62–0.96). In contrast, the RADICALS-HD trial [43] compared different hormonal therapy durations in people receiving postoperative sRT, finding that 24 months of ADT outperformed 6 months in improving MFS (hazard ratio 0.773, 95% CI 0.612–0.975) and consequently delaying the need for salvage ADT. However, when short-term ADT (6 months) was compared to no ADT, the former delayed the time to salvage ADT but did not significantly improved MFS.

Although these findings highlight the benefits of ADT in the BCR setting in general, the optimal duration remains uncertain, in particular for patients with clinically node-positive disease.

Recent and ongoing research has expanded to refine the role of systemic therapy in combination with sRT, particularly after the introduction of androgen receptor pathway inhibitors (ARPI). The aforementioned EMBARK trial [28^▪▪^] showed that enzalutamide, either alone or combined with ADT, significantly prolonged MFS in patients with high-risk BCR that were not candidates for sRT, compared to ADT alone (enzalutamide versus ADT: hazard ratio 0.63, 95% CI 0.46–0.87; enzalutamide+ADT versus ADT: hazard ratio 0.42, 95% CI 0.30–0.61). In the EMBARK trial patients were excluded if they had evidence of distant metastatic disease on conventional imaging, without mentioning the possibility of pelvic nodal involvement. Likewise, the SALV-ENZA trial [44], a randomized phase 2 trial, evaluated men with BCR after radical prostatectomy for high-risk PCa treated with sRT (excluding pelvic nodes) plus 6 months of enzalutamide monotherapy versus sRT alone. The combination proved safe and delayed PSA progression in comparison to sRT alone [freedom from PSA progression (FFPP): 0.42, 95% CI 0.19–0.92); however, these results are still insufficient to warrant a change in clinical practice and pelvic nodes were not included in the sRT field. The FORMULA-509 trial (https://ascopubs.org/doi/10.1200/JCO.2023.41.6_suppl.303; 2023) recruited 345 participants with a detectable PSA post-radical prostatectomy for PCa with unfavorable features (Gleason score 8–10, PSA >0.5, pT3/T4, pN1 or radiographic N1, PSA doubling time <10 months, negative margins, persistent PSA, regional disease, or Decipher High Risk). All patients received sRT (±ENRT, depending on clinical decision) plus 6 months of ADT and randomization was to concurrent bicalutamide 50 mg or abiraterone 1 g + prednisone 5 mg + apalutamide 240 mg. The primary analysis, presented at ASCO 2023, suggests the addition of abiraterone/apalutamide may improve PFS and MFS, particularly in the subgroup of patients with PSA greater than 0.5 ng/ml (3-year PFS: hazard ratio 0.50, 90% CI 0.30–0.86, *P* = 0.03; 3-year MFS: hazard ratio 0.32, 90% CI 0.15–0.72, *P* = 0.01). The final results of the FORMULA-509 trial will report PSA progression-free survival as main outcome, and as secondary endpoints MFS both on conventional and PET imaging.

One of the limitations of these studies is that they did not incorporate PSMA-PET prior to randomization, along with all the considerations we previously discussed; nonetheless, their findings remain valuable in clinical settings where PSMA-PET is not available.

## FUTURE PERSPECTIVES

The introduction of advanced diagnostic tools, such as PSMA-PET, alongside targeted therapeutic strategies and novel systemic treatments, is paving the way for innovative approaches to managing oligorecurrent and oligometastatic PCa. These developments hold promise for optimizing outcomes in patients with limited disease burden. However, definitive guidance on the optimal management of nodal recurrences following radical treatment awaits the results of several ongoing prospective trials: for instance, the OLIGOPELVIS 2 GETUG-P12 (NCT03630666) a phase 3 comparing 6-months ADT with or without ENRT and the POINTER-PC phase 3 trial comparing ENRT vs. SBRT.

Ongoing trials are evaluating the role of intensification of androgen blockade in the landscape of high-risk BCR: PRESTO (NCT03009981) a phase 3 trial comparing ADT, ADT+apalutamide or ADT+apalutamide+abiraterone in men with prior radiation or not candidate to radiotherapy, and PLNs 2 cm or less (lesions identified on PET but invisible on conventional imaging are allowed), CARLHA-2 GETUG-33 (NCT04181203) and PRIMORDIUM (NCT04557059) comparing sRT + ADT with or without apalutamide. These trials are expected to clarify whether treatment intensification with ARPIs can improve disease control and delay progression in men with high-risk BCR, ultimately guiding more personalized therapeutic strategies.

## CONCLUSION

Current guidelines recommend salvage radiotherapy with ADT for pelvic-limited nodal recurrences, with the potential sole use of ENRT or SBRT in highly selected patients enrolled in prospective studies. Evidence suggests that ENRT with simultaneous boost to PSMA-visible nodes, combined with ADT, balances toxicity and disease control. For selected patients with high-risk BCR, enzalutamide with or without ADT could be considered. A multidisciplinary approach involving imaging specialists, pathologists, urologists, medical oncologists, and radiation oncologists is essential for optimizing personalized patient outcomes.

## Acknowledgements

*None.*

### Financial support and sponsorship

*None.*

### Conflicts of interest

*There are no conflicts of interest.*

## REFERENCES AND RECOMMENDED READING

Papers of particular interest, published within the annual period of review, have been highlighted as:

▪ of special interest
▪▪ of outstanding interest
